# The use of kallikrein-related peptidases as adjuvant prognostic markers in colorectal cancer

**DOI:** 10.1038/sj.bjc.6605033

**Published:** 2009-04-14

**Authors:** M Talieri, L Li, Y Zheng, D K Alexopoulou, A Soosaipillai, A Scorilas, D Xynopoulos, E P Diamandis

**Affiliations:** 1 Department of Cellular Physiology, ‘G. Papanicolaou’ Research Center of Oncology, ‘Saint Savvas’ Hospital, 171 Alexandras Avenue, Athens 11522, Greece; 2 Department of Biostatistics and Biomathematics, Fred Hutchinson Cancer Research Center, North Seattle WA 98109-1024, USA; 3 Department of Pathology and Laboratory Medicine, Mount Sinai Hospital and University of Toronto, 60 Murray Street, 6th Floor, Room L6-2001, Toronto, ON, Canada M5T 3L9; 4 Department of Biochemistry and Molecular Biology, Faculty of Biology, University of Athens, Panepistimioupolis, Athens 15701, Greece; 5 Department of Gastroenterology, ‘Saint Savvas’ Hospital, 171 Alexandras Avenue, Athens 11522, Greece

**Keywords:** colorectal cancer, ELISA, kallikrein-related peptidases, KLKs, tumour markers

## Abstract

Several members of the human tissue kallikrein-related peptidase (KLK) family are emerging cancer biomarkers. The aim of this study was to analyse the expression of a panel of KLKs in colorectal cancer and to find out if the multiparametric combination of them can increase the accuracy of prediction of patients survival beyond the traditional clinical information. Nine KLKs (KLK5-8, KLK10, KLK11, KLK13-15) were measured using ELISA assays in cytosolic extracts of 122 colon cancer tissues and their nearby normal mucosa, obtained during surgery. The mean levels of almost all KLKs in tumour tissues were significantly different from their counterparts of normal tissue (*P*<0.0001). KLK 5, 6, 7, 13, 14 were significantly associated with overall survival in univariate analysis, but after adjusting for age, TNM and differentiation stage, only KLK5 (HR: 1.24 (95% CI: 1.05–1.47)), KLK7 (HR: 1.57 (95% CI: 1.04–2.37)) and KLK14 (HR: 1.43 (95% CI: 1.05–1.94)) remained significant. Addition of a panel of selected KLK markers to clinical parameters gave an increment in AUC of 0.86 beyond the clinical factors at year 1, showing that it can increase the accuracy of prediction of overall survival beyond the traditional clinical information, particularly the short-term (1 year) survival after surgery.

Colorectal cancer (CRC) is the cause of half a million deaths worldwide every year. There are also about 1 million new cases diagnosed annually, making it the third most common type of cancer in the world, despite the fact that it mostly affects populations of western lifestyle (American Cancer Society, 2005; McCracken *et al*, 2007). The variation in cancer survival within the same countries depends on the socio-economic level of the people affected (Coleman *et al*, 2008). In the past few years, the death rate caused by CRC has been reduced, mainly because of increased screening based on serum markers, such as carcinoembryonic antigen (Sturgeon, 2002), endoscopic technology and improved treatment of the disease (Duffy *et al*, 2003). Tissue-based markers have been proposed as possible prognostic markers and predictors of response to treatment. Such markers include thymidilate synthase, the transcription factor p53, the oncogene K-ras and the adenomatous polyposis coli gene. Nevertheless, there are remarkable contradictions in the literature with regard to the relation between common CRC genes and prognosis (Anwar *et al*, 2004). Therefore, extensive research is needed to blend current endoscopic and surgical technology with specific molecular markers that will discriminate subgroups of patients for prognosis or specific targeted therapies.

Proteases may represent good diagnostic/prognostic biomarkers, as they are involved in cancer progression (Duffy, 1996). Human tissue kallikrein-related peptidases (KLKs) are a family of 15 serine proteases with diverse physiological functions. KLKs play important roles in different physiologic processes such as regulation of cell growth and differentiation, tissue remodelling, angiogenesis (Borgono and Diamandis, 2004), skin desquamation, human semen liquefaction (Pampalakis and Sotiropoulou, 2007), dental enamel formation (Lu *et al*, 2008), neuro-degeneration (Scarisbrick *et al*, 2008), inflammation (Oikonomopoulou *et al*, 2007), cervico-vaginal physiology (Shaw and Diamandis, 2008), and vascularisation (Smith *et al*, 2008). So far, KLKs have been widely examined as cancer biomarkers, mostly in steroid-hormone-regulated cancers (Emami and Diamandis, 2007), because steroid hormones play an important role in the regulation of kallikrein transcription (Paliouras *et al*, 2007). Many members of the kallikrein family have been reported to be promising diagnostic/prognostic biomarkers for several cancer types, including breast, ovarian, prostate and testicular carcinomas (Borgono and Diamandis, 2004). However, recent studies indicate that other mechanisms probably cooperate in *KLK* regulation, including the use of alternative promoters (Christophi *et al*, 2004), the production of multiple splice variants (Pampalakis *et al*, 2006) or epigenetic alterations, like DNA methylation and histone modification (Pampalakis *et al*, 2004; Emami and Diamandis, 2007). Kallikrein-related peptidases may also be involved in cancer pathogenesis by degrading extracellular matrix proteins or promoting angiogenesis (Borgono and Diamandis, 2004). Few studies in non-hormone regulated cancers have been conducted so far concerning lung (Petraki *et al*, 2006; Planque *et al*, 2008), pancreatic (Yousef *et al*, 2004; Dong *et al*, 2008), head and neck (Chung *et al*, 2004) and brain (Prezas *et al*, 2006b) cancers and leukaemia (Roman-Gomez *et al*, 2004) and their relation with KLKs. Until now, only KLK6 and KLK10 have been examined as biomarkers in CRC by Ogawa *et al* (2005) and Feng *et al* (2006) using real time PCR and by Yousef *et al* (2004) using *in silico* analysis. The aim of this study was to evaluate whether human tissue KLKs, alone or in combination with established clinical and pathological variables in a multiparametric model, can be used as potential prognostic markers for CRC patients.

## Materials and methods

### Clinical samples

Primary colorectal cancer specimens from 122 patients, collected at the Oncologic Hospital of Athens ‘Saint Savvas’, were staged according to Dukes' operative staging system. Paired normal colon mucosa, sufficiently separated from the tumours, was available for all cancer samples. Both cancer and normal samples were histologically confirmed by eosin–haematoxylin staining. Investigations were carried out in accordance with the ethical standards of the Helsinki Declaration of 1975, as revised in 1983. Histologic diagnoses and grading of tumours were made based on the revised World Health Organization (WHO) classification for colon tumours. All cases under study came from complete surgical excision of the tumour. No chemotherapy or radiotherapy was administered before surgery. Clinical and pathological characteristics such as age, tumour size, TNM stage, tumour grade and differentiation, were available for all patients. Two time-to-event outcomes after surgery were recorded: Disease-Free Survival (DFS) and Overall Survival (OS). DFS in each case was defined as the time interval between the date of surgical removal of the primary cancer and the date of the first documented evidence of relapse. OS was defined as the time interval between the date of surgery and the date of death, or the date of last follow up for those who were alive at the end of the study.

### Ethics

Agreement of the Institute's Ethics Committee for the scientific analysis of tumour tissues, as well as patients' written informed consent had been obtained.

### Preparation of colon tissue extracts

Upon collection, colon tissues were snap frozen in liquid nitrogen and subsequently transferred to −80°C until extraction. Frozen samples (0.2 g) were pulverised using a Mikro-Dismembrator – *U* (Sartorius, Goettingen, Germany) and processed as described earlier (Shaw and Diamandis, 2007). Protein concentration of the extracts was determined (Pierce Chemical Co, Rockford, IL, USA).

### Immunofluorometric ELISA measurement of kallikreins in colorectal cytosolic extracts

Concentration of KLKs was measured with a non-competitive immunoassay, described earlier (Shaw and Diamandis, 2007; Planque *et al*, 2008). Two types of configurations of ELISA were used in this study, using either monoclonal–monoclonal (KLK5, KLK6, KLK7, KLK8, KLK10, KLK13) or monoclonal–polyclonal (KLK11, KLK12, KLK14) combinations. All ELISAs were tested negative for cross-reactivity against other KLKs.

### Statistical methods

Marker measurements were logarithmically transformed due to skewness in the distributions. The differentiation in marker expression in paired tumour and normal tissues was evaluated by the paired *t*-test on the transformed values. The relationships between biomarkers and tumour and patient characteristics were examined with the Kruskal–Wallis test. Spearman's rank correlation coefficient was used to assess the correlations among biomarkers (Supplementary Table S1 along with hierarchical clustering based on Spearman's correlation). Cox regression model was applied to evaluate the hazard ratios (HR) of biomarkers on DFS or OS applying univariate or multivariate analyses. Clinical variables, including age, tumour size, TNM stage, grade and tumour differentiation were adjusted in multivariate Cox proportional hazards models. Both HRs and 95% confidence interval (95% CI) were calculated on log-transformed biomarkers and were represented with two-sided *P*-values. In this study, to further evaluate the prognostic usefulness of the markers, we used a receiver operating characteristic (ROC) curve (Heagerty *et al*, 2000). ROC analysis was first conducted on individual markers and then on their combination to explore the possibility of a marker panel to lead to improved performance. We used an algorithm that gives a single composite score in the sense that the ROC curve is maximised at every threshold value. To get a prognostic index we built marker panels at year 1 and 5 after the surgery using a weighted logistic regression that is appropriate for censored failure time (Zheng *et al*, 2006) with the stepwise selection. The predictive accuracy of composite scores was evaluated based on a re-sampling algorithm to correct for potential overfitting when deriving the combinatory rule (Supplementary Figure S1). Specifically, we randomly split data into a training set and a validation set. The training set included two thirds of the observations, and the validation set included one third of the observations. Using the training set, we first operated a model selection from which the final selected model gave rise to the linear combination rule. We then, calculated two ROC curves for the linear score: one with using data from the training set and the other using the validation set. The vertical differences between the two ROC curves gave the overestimation of the sensitivities at a given specification. The whole procedure was repeated 100 times, and these differences were averaged to yield an estimate of the expected overestimation. We present both the original ROC curves and the ROC curves that are corrected for overestimation. All analyses were done using Statistical Analysis System (SAS Institute Inc., Cary, NC, USA) and S-plus 7.0 software (Insightful Corp., Palo Alto, CA, USA).

## Results

### Distribution of kallikreins between colon cancer and its paired normal tissues

To determine the clinical utility of the kallikrein family as potential tumour markers for colorectal cancer, the expression profile of several members of the family was examined by immunofluorometric ELISA assays, previously developed and validated (Dorn *et al*, 2007; Shaw and Diamandis, 2007; Planque *et al*, 2008). The detection limit was 0.05 mg l^−1^ for KLK5, KLK6, KLK10, KLK13, KLK14 and KLK15, but only 0.2 mg l^−1^ for KLK7, KLK8 and KLK11. Table 1 shows the distribution of numerical variables of the study. The differential expression of KLK5-8, KLK10, KLK11 and KLK13-15 was evaluated by the paired *t*-test on log-transformed values. As shown in Table 2, the means of kallikrein concentrations between cancer and normal samples were significantly different for all KLKs (*P*<0.001), with the exception of KLK5 (*P*=0.92) and KLK14 (*P*=0.87).

### Association of kallikrein markers with overall survival of colon cancer patients

Cox regression models were used to examine the association between OS and kallikreins, and clinical and pathological parameters (Table 3). In univariate analysis the clinical and pathological parameters, age, grade, TNM stage and tumour differentiation, are significantly associated with OS (*P*=0.001, *P*=0.014, *P*<0.001 and *P*=0.004, respectively), whereas in multivariate analysis only age, TNM stage and tumour differentiation remained significant (*P*=0.005, *P*=0.004 and *P*=0.036, respectively). Kallikrein markers KLK5, KLK6, KLK7, KLK13, and KLK14 were significantly associated with OS (*P*⩽0.05) in univariate analysis, but after adjusting for the significant clinical factors, age, TNM stage and differentiation, only KLK5 (HR: 1.24 (95% CI: 1.05–1.47)), KLK7 (HR: 1.57 (95% CI: 1.04–2.37)) and KLK14 (HR: 1.43 (95% CI: 1.05–1.94)) remained significant (Table 3). Whereas, when using the forward selection with a *P*-value of 0.1 as entry criterion in the Cox regression, only KLK14 and KLK7 were selected in the multi-marker model. The fitted Cox model is: 0.054 × Age+1.629 × TNM (Stage III/IV)+0.703 × Differentiation (poor)+0.300 × logKLK14+0.364 × logKLK7.

**Figure 1:**
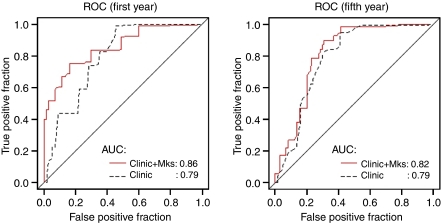
ROC curves for 1- and 5-year overall survival for ‘Clinical’: age and TNM stage; and ‘Clinical+Markers’: age, TNM stage, and selected marker panels of KLK(s). The marker panel at year 1 is: KLK14. The marker panel at year 5 is: KLK8, KLK10, KLK14, and KLK15.

The clinical usefulness of kallikreins for prediction of OS status at 1 year and 5 years after operation for CRC was evaluated by ROC curve analysis. For OS, age and TNM stage alone were very predictive with an area under the curve (AUC) of 0.79 at both year 1 and year 5 under the time-dependent ROC curve. However, the addition of a panel of selected KLK markers (KLK14) at year 1 by stepwise selection in weighted logistic regression, gave an increment in AUC from 0.79 to 0.86, but much less at year 5 (Figure 1).

### Association of kallikrein markers with disease-free survival of colon cancer patients

**Figure 2:**
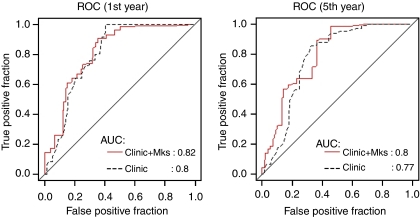
ROC curves for 1- and 5-year disease-free survival for ‘Clinical’: age and TNM stage; and ‘Clinical+Markers’: age, TNM stage, and selected marker panels of KLK(s). The marker panel at year 1 is: KLK10 and KLK14. The marker panel at year 5 is: KLK5, KLK6, KLK8, KLK10, KLK13, KLK14, and KLK15.

Cox regression model results for colorectal cancer DFS are listed in Table 4. Among all clinical and pathological parameters, only TNM stage had a significant (*P*=0.04) hazard ratio in multivariate analysis. Kallikrein markers KLK5, KLK6, KLK7, KLK10, KLK13, and KLK14 were significantly associated with DFS (*P*⩽0.05) in univariate analysis, but after adjusting for TNM stage only KLK14 (HR: 1.33 (95% CI: 1.05–1.68)) remained significant and no other marker entered into the multivariate Cox model using the forward selection with a *P*-value of 0.1 as entry criterion. The fitted Cox model is: 1.404 × TNM (Stage III/IV)+0.283 × logKLK14. The clinical usefulness of kallikreins for the prediction of DFS status at 1 year and 5 years after operation for colorectal cancer was also evaluated by ROC curve analysis. The addition of a panel of selected KLK markers by stepwise selection in weighted logistic regression hardly gave any increment values at both year 1 and year 5 (Figure 2).

## Discussion

Tissue or serum levels of KLKs have been examined either individually or in panels as diagnostic or prognostic factors in different types of cancer (Borgono and Diamandis, 2004; Dorn *et al*, 2007; Paliouras *et al*, 2007; Zheng *et al*, 2007; Planque *et al*, 2008). With the development of technology and automation and the promising concept of individualised therapy, it is tempting to examine concurrently, in panels, many different parameters as cancer biomarkers. In this study, we analysed the cytosolic extracts of 122 pairs of cancer/normal colon mucosa for the expression of nine KLKs on the protein level, to evaluate the clinical utility of this gene family as prognostic markers for colorectal cancer. Using a sensitive and specific immunofluorometric assay developed at Professor Diamandis's laboratory (University of Toronto, Ontario, Canada), we studied the alterations in the expression between cancer and paired normal colorectal tissue for nine KLKs. In addition, we used extensive statistical analysis to examine whether the use of those KLKs, as a panel, has to offer more information to the prognosis of colorectal cancer patients than the already existent clinical and pathological parameters.

Almost all KLKs, except KLKs 5 and 14, effectively separate cancer from paired normal tissue in a statistically significant manner. This is in agreement with our unpublished data showing overexpression of several kallikreins (*KLK6*, *KLK7*, *KLK10*) on the mRNA level of cancer samples as compared with their paired normal colon mucosa. Similar studies using the same method with us and examining the expression patterns of several kallikreins either in ovarian cytosolic extracts (Dorn *et al*, 2007; Zheng *et al*, 2007) or serum samples from lung cancer patients (Planque *et al*, 2008), have revealed unique patterns.

The present report confirms the *in silico* study by Yousef *et al*, 2004, showing an overexpression of KLK6, KLK8 and KLK10 in CRC. It is also in agreement with Ogawa *et al* (2005), reporting that *KLK6* mRNA overexpression in CRC correlates with poor prognosis of CRC patients. Furthermore, we are in line with Feng *et al* (2006), reporting upregulation of *KLK10* in CRC. We underline as important the difference noticed between the upregulation of KLK10 in CRC shown in this work and its downregulation shown in previous studies and attributing it to CpG island hypermethylation, examining other cancer tissue types, like breast, prostate or ovarian (Sidiropoulos *et al*, 2005), gastric (Huang *et al*, 2007), head and neck (Worsham *et al*, 2006), testicular (Luo *et al*, 2001) and lung (Planque *et al*, 2008). However, contradictory results have been reported in other studies, claiming that KLK10 is upregulated in cancers, as breast (Luo *et al*, 2002), ovarian (Luo *et al*, 2003), gastric (Feng *et al*, 2006) and head and neck (Dasgupta *et al*, 2006).

All members of the KLK family have been examined as biomarkers for different types of cancer and reviewed recently by Paliouras *et al*, 2007. Current results showing an overexpression of KLK6 in CRC may be explained by the fact that KLK6 acts as a mediator of K-Ras-dependent migration of CRC cells, as reported by Henkhaus *et al* (2008a) and that K-RAS mutations are common in CRC, occurring in approximately 50% of cases. We have recently demonstrated that ovarian cancer cells of epithelial origin like colon cells, stably transfected to express KLKs 4, 5, 6 and 7 were significantly more invasive *in vitro* and formed larger tumours in mice (Prezas *et al*, 2006a). Furthermore, Klucky *et al*, 2007 trying to explain the function of KLK6 in cancer, provided evidence that KLK6 induces E-cadherin shedding and thus, promotes cell proliferation, migration, and invasion. In addition, recent data presented by Henkhaus *et al* (2008b) indicate a central role for caveolin-1, the main structural protein of caveolae in both KLK6 gene expression and protein secretion of colon cancer cell line HCT116.

Cytosolic KLK7 protein in this study and *KLK7* transcripts from the same samples used in an earlier study conducted in our lab (Talieri *et al*, 2009), seem to follow the same pattern of upregulation. As reported recently by Ramani and Haun (2008), overexpression of KLK7 in tumour cells may play an important role in tumour invasion through proteolysis of extracellular matrix components, such as fibronectin. Our lab has shown in the past that *KLK7* may serve as a potential biomarker for breast cancer patients (Talieri *et al*, 2004) and that its overexpression in intracranial tumours reveals a less favourable outcome (Prezas *et al*, 2006b). Moreover, we have shown that U-251-MG glioblastoma cells transfected with *KLK7* showed increased invasive potential in an *in vitro* Matrigel assay (Prezas *et al*, 2006b). Present work shows upregulation of KLK8, although it is not associated either with OS or DFS. This is in line with our previous work (Prezas *et al*, 2006b) in brain cancer, where we did not find any association of KLK8 with DFS or OS. In addition, in the same study, glioblastoma U-251-MG cells stably transfected by the *KLK8* gene did not show invasion in the Matrigel assay. Although the expression of KLK14 of CRC samples does not seam to be significantly different from their normal counterparts, it is the marker that remained statistically significant in association with the survival outcomes: DFS and OS in the Cox regression univariate and multivariate analysis showing unfavourable prognosis for the patients. KLK14 is involved in extracellular matrix degradation (Borgono *et al*, 2007) and the present finding may be associated with response to treatment.

As most members of the KLK family display differential expression in CRC, it is very tempting to speculate on their potential role in CRC initiation and progression. Studies on the physiological function of KLKs in normal colon will shed more light into the roles of these enzymes in colon cancer and other diseases of colon and rectum. In addition, these enzymes may represent promising future therapeutic targets.

We need to point out that even though a combination of selected clinical factors and KLKs panel showed an increase in OS prediction at 1 year after the surgery with AUC of 0.86 under the time-dependent ROC curve, after correction for possible overfitting from the model selection process estimated by cross-validation, the corrected AUC was only at 0.69 and it was no better than the prediction of age and TNM stage alone (Supplementary Figure S1). Therefore, the performance of this selected KLK marker panel and the combination rule defined in this particular data need to be further evaluated in an independent validation study.

## Figures and Tables

**Table 1:** Distribution of numerical variables of the study

			**Quartiles**		
			**25**	**50**	**75**
**Variable**	**Mean±s.e.**	**Range**	**(median)**		
Patient age (years)	68.7±0.8	31.0–91.0	61.7	70.0	76.0
Tumour size (cm)	4.7±0.1	0.2–12.0	3.5	4.5	5.5
Follow-up (months; *n*=122)	37.2±2.5	1.0–132.0	10.0	35.5	54.7
DFS (months; *n*=122)	31.3±2.7	0.0–132.0	4.2	24.0	54.0
OS (months; *n*=122)	37.1±2.5	1.0–132.0	10.0	35.5	54.7

DFS=disease-free survival; OS=overall survival.

**Table 2:** Marker distribution in 122 paired tumour (1) and normal tissues (0) and the distribution of the expression ratios of tumour to normal

**Marker**	**CA/normal**	**Median (1st, 3rd quartile)**	**Mean ratio^a^ (s.e.)**	**95% CI**	*P*-value^**^
KLK5	1	0.005 (0.004, 0.008)	0.985 (0.146)	0.736–1.318	0.9198
	0	0.006 (0.005, 0.01)			.
KLK6	1	0.4485 (0.104, 1.684)	15.548 (2.47)	11.388–21.228	<0.0001
	0	0.0315 (0.01, 0.06)			.
KLK7	1	0.29 (0.171, 0.51)	2.323 (0.235)	1.905–2.832	<0.0001
	0	0.13 (0.078, 0.217)			.
KLK8	1	0.009 (0.005, 0.023)	2.001 (0.221)	1.611–2.485	<0.0001
	0	0.0055 (0.004, 0.008)			.
KLK10	1	0.464 (0.071, 2.206)	33.307 (6.398)	22.857–48.535	<0.0001
	0	0.008 (0.005, 0.029)			.
KLK11	1	0.817 (0.291, 1.98)	3.131 (0.435)	2.385–4.11	<0.0001
	0	0.266 (0.15, 0.568)			.
KLK13	1	0.009 (0.005, 0.016)	1.518 (0.148)	1.254–1.838	<0.0001
	0	0.005 (0.004, 0.008)			.
KLK14	1	0.035 (0.019, 0.061)	0.98 (0.123)	0.767–1.253	0.8738
	0	0.043 (0.032, 0.064)			.
KLK15	1	0.132 (0.063, 0.256)	3.263 (0.512)	2.4–4.438	<0.0001
	0	0.0365 (0.006, 0.14)			.

CI=confidence interval; KLK=kallikrein-related peptidase.

a Back-transformed mean log ratios of cancer to normal expressions.

^**^P-values from the paired *t*-test on log-transformed values.

**Table 3:** The univariate and multivariate Cox regression analysis for the overall survival

		**Univariate**			**Multivariate**		
**Clinical variable**	***N* (%)**	**HR**	**CI**	*P* ^*^	**HR**	**CI**	*P-*value^**^
Age (years)	123	1.06	(1.02, 1.09)	0.001	1.07	(1.02, 1.12)	0.004
Tumour size	116	1.01	(0.84, 1.22)	0.911	1.07	(0.73, 1.56)	0.721
							
*Grade*							
I or II	65 (68)	1			1		
III or IV	30 (32)	2.4	(1.19, 4.82)	0.014	0.57	(0.17, 1.88)	0.356
							
*TNM stage*							
I or II	59 (51)	1			1		
III or IV	56 (49)	4.39	(2.08, 9.26)	<0.001	4.71	(1.62, 13.66)	0.004
							
*Differentiation*							
Well-moderate	99 (86)	1			1		
Poor	16 (14)	2.92	(1.41, 6.02)	0.004	3.7	(1.09, 12.6)	0.036
							
*Markers*							
KLK5	126	1.25	(1.1, 1.42)	0.001	1.24	(1.05, 1.47)	0.012
KLK6	127	1.26	(1.03, 1.54)	0.026	1.09	(0.85, 1.39)	0.493
KLK7	127	1.78	(1.32, 2.4)	<0.001	1.57	(1.04, 2.37)	0.032
KLK8	127	1.1	(0.91, 1.34)	0.335	1.01	(0.78, 1.32)	0.914
KLK10	127	1.15	(1, 1.33)	0.051	1.12	(0.94, 1.34)	0.193
KLK11	126	1.2	(0.99, 1.46)	0.057	1.16	(0.91, 1.49)	0.236
KLK13	127	1.31	(1.03, 1.68)	0.028	1.36	(1, 1.87)	0.053
KLK14	127	1.32	(1.1, 1.6)	0.003	1.43	(1.05, 1.94)	0.022
KLK15	127	1.01	(0.78, 1.32)	0.934	1.02	(0.77, 1.36)	0.891

CI=confidence interval; KLK=kallikrein-related peptidase.

^*^P-values from univariate Cox regression.

^**^P-values for clinical variables are from multivairate Cox regression after adjusting for each other.

*P*-values for markers are from multiviariate Cox regression with significant clinical variables from above and the marker of interest.

**Table 4:** The univariate and multivariate Cox regression analysis for the disease-free survival

		**Univariate**			**Multivariate**		
**Clinical variable**	***N* (%)**	**HR**	**CI**	*P* ^*^	**HR**	**CI**	*P-*value^**^
Age (years)	124	1.04	(1.01, 1.07)	0.013	1.03	(0.99, 1.08)	0.101
Tumour size	116	1.01	(0.84, 1.21)	0.951	0.94	(0.68, 1.31)	0.721
							
*Grade*							
I or II	65 (68)						
III or IV	31 (32)	3.04	(1.55, 5.97)	0.001	1.33	(0.45, 3.95)	0.605
							
*TNM stage*							
I or II	59 (51)						
III or IV	57 (49)	4.1	(2.01, 8.39)	0.000	2.86	(1.05, 7.79)	0.040
							
*Differentiation*							
Well-moderate	99 (85)						
Poor	17 (15)	2.78	(1.42, 5.43)	0.003	2.04	(0.72, 5.81)	0.182
							
*Markers*							
KLK5	127	1.21	(1.07, 1.37)	0.002	1.16	(0.99, 1.37)	0.065
KLK6	128	1.23	(1.03, 1.48)	0.024	1.09	(0.9, 1.33)	0.384
KLK7	128	1.47	(1.14, 1.9)	0.003	1.28	(0.96, 1.72)	0.095
KLK8	128	1.09	(0.91, 1.31)	0.349	1	(0.8, 1.26)	0.972
KLK10	128	1.15	(1.01, 1.31)	0.040	1.07	(0.92, 1.23)	0.387
KLK11	127	1.18	(0.99, 1.41)	0.068	1.12	(0.9, 1.39)	0.298
KLK13	128	1.32	(1.04, 1.67)	0.025	1.29	(0.98, 1.71)	0.074
KLK14	128	1.28	(1.06, 1.54)	0.011	1.33	(1.05, 1.68)	0.018
KLK15	128	1.07	(0.84, 1.37)	0.584	1.12	(0.85, 1.46)	0.425

CI=confidence interval; KLK=kallikrein-related peptidase.

^*^P-values from univariate Cox regression.

^**^P-values for clinical variables are from multivairate Cox regression after adjusting for each other.

*P*-values for markers are from multiviariate Cox regression with clinical variable TNM stage and the marker of interest.

## References

[bib1] American Cancer Society (2005) Cancer Facts and Figures, 2005. American Cancer Society: Atlanta

[bib2] Anwar S, Frayling IM, Scott NA, Carlson GL (2004) Systematic review of genetic influences on the prognosis of colorectal cancer. Brit J Surg 91: 1275–1291 15382104 10.1002/bjs.4737

[bib3] Borgono CA, Diamandis EP (2004) The emerging roles of human kallikreins in cancer. Nat Rev Cancer 4: 876–890 15516960 10.1038/nrc1474

[bib4] Borgono CA, Michael IP, Show JLV, Luo L-Y, Ghosh MC, Soosaipillai A, Grass L, Katsaros D, Diamandis EP (2007) Expression and functional characterization of the cancer-related serine protease, human tissue kallikrein 14. J Biol Chem 282: 2405–2422 17110383 10.1074/jbc.M608348200

[bib5] Christophi GP, Isackson PJ, Blaber S, Blaber M, Rodriquez M, Scarisbrick IA (2004) Distinct promoters regulate tissue-specific and differential expression of kallikrein 6 in CNS demyelinating disease. J Neurochem 91: 1439–1449 15584920 10.1111/j.1471-4159.2004.02826.x

[bib6] Chung CH, Parker JS, Karaca G, Wu J, Funkhouser WK, Moore D, Butterfoss D, Xiang D, Zanation A, Yin X, Shockley WW, Weissler MC, Dressler LG, Shores CG, Yarbrough WG, Perou CM (2004) Molecular classification of head and neck squamous cell carcinomas using patterns of gene expression. Cancer Cell 5: 489–500 15144956 10.1016/s1535-6108(04)00112-6

[bib7] Coleman MP, Quaresma M, Berrino F, Lutz J-M, De Angelis R, and the CONCORD Working Group (2008) Cancer survival in five continents: a worldwide population-based study(CONCORD). Lancet Oncol 9: 730–756 18639491 10.1016/S1470-2045(08)70179-7

[bib8] Dasgupta S, Tripathi PK, Qin H, Bhattacharya-Chatterjee M, Valentino J, Chatterjee SK (2006) Identifiction of molecular targets for immunotherapy of patients with head and neck squamous cell carcinoma. Oral Oncol 42: 306–316 16321566 10.1016/j.oraloncology.2005.08.007

[bib9] Dong Y, Matigian N, Harvey TJ, Samaratunga H, Hooper JD, Clements JA (2008) Tissue-specific promoter utilisation of the kallikrein-related peptidase genes, KLK5 and KLK7, and cellular localisation of the encoded proteins suggest roles in exocrine pancreatic function. Biol Chem 389: 99–109 18163887 10.1515/BC.2008.013

[bib10] Dorn J, Schmitt M, Kates R, Schmalfeldt B, Kiechle M, Scorilas A, Diamandis EP, Harbeck N (2007) Primary tumor levels of human tissue kallikreins affect surgical success and survival in ovarian cancer patients. Clin Cancer Res 13: 1742–1748 17363527 10.1158/1078-0432.CCR-06-2482

[bib11] Duffy MJ (1996) Proteases as prognostic markers of cancer. Clin Cancer Res 2: 613–618 9816210

[bib12] Duffy MJ, van Dalen A, Haglund C, Hansson L, Klapdor R, Lamerz R, Nilsson O, Sturgeon C, Topolcan O (2003) Clinical utility of biochemical markers in colorectal cancer: European Group on Tumour Markers (EGTM) guidelines. Eur J Cancer 39: 718–727 12651195 10.1016/s0959-8049(02)00811-0

[bib13] Emami N, Diamandis EP (2007) Human tissue kallikreins: a road under construction. Clin Chim Acta 381: 78–84 17382920 10.1016/j.cca.2007.02.023

[bib14] Feng B, Xu WB, Zheng MH, Ma JJ, Cai Q, Zhang Y, Ji J, Lu AG, Qu Y, Li JW, Wang ML, Hu WG, Liu BY, Zhu ZG (2006) Clinical significance of human kallikrein 10 gene expression in colorectal cancer and gastric cancer. J Gastroenterol Hepatol 21: 1596–1603 16928223 10.1111/j.1440-1746.2006.04228.x

[bib15] Heagerty PJ, Lumley T, Pepe MS (2000) Time-dependent ROC curves for censored survival data and a diagnostic marker. Biometrics 56: 337–344 10877287 10.1111/j.0006-341x.2000.00337.x

[bib16] Henkhaus RS, Gerner EW, Ignatenko NA (2008a) Kallikrein 6 is a mediator of K-RAS-dependent migration of colon carcinoma cells. Biol Chem 389: 757–764 18627290 10.1515/BC.2008.087 PMC3574817

[bib17] Henkhaus R, Roy UKB, Cavallo-Medved D, Sloane BF, Gerner EW, Ignatenko NA (2008b) Caveolin-1-Mediated Expression and Secretion of kallikrein 6 in colon cancer cells. Neoplasia 10: 140–148 18283336 10.1593/neo.07817 PMC2244689

[bib18] Huang W, Zhong J, Wu L-Y, Yu L-F, Tian X-l, Zhang Y-F, Li B (2007) Downregulation and CpG island hypermethylation of *NES1/hK10* gene in the pathogenesis of human gastric cancer. Cancer Lett 251: 78–85 17182177 10.1016/j.canlet.2006.11.006

[bib19] Klucky B, Mueller R, Vogt I, Teurich S, Hartenstein B, Breuhahn K, Flechtenmacher Ch, Angel P, Hess J (2007) Kallikrein 6 induces E-cadherin shedding and promotes cell proliferation, migration, and invasion. Cancer Res 67: 8198–8206 17804733 10.1158/0008-5472.CAN-07-0607

[bib20] Lu Y, Papagerakis P, Yamakoshi Y, Hu JCC, Bartlett JD, Simmer JP (2008) Functions of KLK4 and MMP-20 in dental enamel formation. Biol Chem 389: 695–700 18627287 10.1515/BC.2008.080 PMC2688471

[bib21] Luo LY, Diamandis EP, Look MP, Soosaipillai AP, Foekens JA (2002) Higher expression of human kallikrein 10 in breast cancer tissue predicts tamoxifen resistance. Br J Cancer 86: 1790–1796 12087468 10.1038/sj.bjc.6600323 PMC2375391

[bib22] Luo LY, Katsaros D, Scorilas A, Fracchioli S, Bellino R, van Gramberen M, de Bruijn H, Henrik A, Stenman UH, Massobrio M, van der Zee AG, Vergote I, Diamandis EP (2003) The serum concentration of human kallikrein 10 represents a novel biomarker for ovarian cancer diagnosis and prognosis. Cancer Res 63: 807–811 12591730

[bib23] Luo LY, Rajpert De Meyts ER, Jung K, Diamandis EP (2001) Expression of the normal epithelial cell-specific I (*NES1; KLK10*) candidate tumor suppressor gene in normal and malignant testicular tissue. Br J Cancer 85: 220–224 11461080 10.1054/bjoc.2001.1870 PMC2364047

[bib24] McCracken M, Olsen M, Chen MS, Jemal A, Thun M, Cokkinides V, Deapen D, Ward E (2007) Cancer incidence, mortality, and associated risk factors among Asian Americans of Chinese, Filipino, Vietnamese, Korean, and Japanese ethnicities. CA Cancer J Clin 57: 190–205 17626117 10.3322/canjclin.57.4.190

[bib25] Ogawa K, Utsunomiya T, Mimori K, Tanaka F, Inoue H, Nagahara H, Mori M (2005) Clinical significance of human kallikrein gene 6 messenger RNA expression in colorectal cancer. Clin Cancer Res 11: 2889–2893 15837738 10.1158/1078-0432.CCR-04-2281

[bib26] Oikonomopoulou K, Hansen KK, Chapman K, Vergnolle N, Diamandis EP, Hollenberg MD (2007) Kallikrein-mediated activation of PARs in inflammation and nociception. Inflamm Res 56(Suppl 3): S499–S502

[bib27] Paliouras M, Borgono C, Diamandis E (2007) Human tissue kallikreins: the cancer biomarker family. Cancer Lett 249: 61–79 17275179 10.1016/j.canlet.2006.12.018

[bib28] Pampalakis G, Diamandis E, Sotiropoulou G (2006) The epigenetic basis for the aberrant expression of kallikreins in human cancers. Biol Chem 387: 795–799 16800742 10.1515/BC.2006.100

[bib29] Pampalakis G, Kurlender L, Diamandis EP, Sotiropoulou G (2004) Cloning and chracterization of novel isoforms of the human kallikrein 6 gene. Biochem Biophys Res Commun 320: 54–61 15207701 10.1016/j.bbrc.2004.04.205

[bib30] Pampalakis G, Sotiropoulou G (2007) Tissue kallikrein proteolytic cascade pathways in normal physiology and cancer. Biochimica et Biophysica Acta 1776: 22–31 17629406 10.1016/j.bbcan.2007.06.001

[bib31] Petraki CD, Papanastasiou PA, Karavana VN, Diamandis EP (2006) Cellular distribution of human tissue kallikreins: immunohistochemical localization. Biol Chem 387: 653–663 16800726 10.1515/BC.2006.084

[bib32] Planque Ch, Li L, Zheng Y, Soosaipillai A, Reckamp K, Chia D, Diamandis EP, Goodglick L (2008) A multiparametric serum kallikrein panel for diagnosis of non-small cell lung carcinoma. Clin Cancer Res 14: 1355–1362 18316555 10.1158/1078-0432.CCR-07-4117

[bib33] Prezas P, Arlt MJ, Viktorov P, Soosaipillai A, Holzscheiter L, Schmitt M, Talieri M, Diamandis EP, Krüger A, Magdolen V (2006a) Overexpression of the human tissue kallikrein genes KLK4, 5, 6 and 7 increased the malignant phenotype of ovarian cancer cells. Biol Chem 387: 807–811 16800744 10.1515/BC.2006.102

[bib34] Prezas P, Scorilas A, Yfanti C, Viktorov P, Agnanti N, Diamandis EP, Talieri M (2006b) Human tissue Kallikreins 7 and 8 gene expression in intracranial tumors: a clinical study in Greece. Biol Chem 387: 613–618 10.1515/BC.2006.200 17132107

[bib35] Ramani VC, Haun RS (2008) The extracellular matrix protein fibronectin is a substrate for kallikrein 7. Biochem Biophys Res Commun 369: 1169–1173 18343220 10.1016/j.bbrc.2008.03.021

[bib36] Roman-Gomez J, Jimenez-Velasco A, Agerre X, Castillejo JA, Barrios M, Andreu EJ, Prosper F, Heiniger A, Torres A (2004) The normal epithelial cell-specific 1 (NES1) gene, a candidate tumor suppressor gene on chromosome 19q13.3–4, is downregulated by hypermethylation in acute lymphoblastic leukemia. Leukemia 18: 362–365 14628074 10.1038/sj.leu.2403223

[bib37] Scarisbrick IA, Limbo R, Vandell AG, Keegan M, Blaber SI, Blaber M, Sneve D, Lucchinetti CF, Rodriguez M, Diamandis EP (2008) Kallikreins are associated with secondary progressive multiple sclerosis and promote neurodegenaration. Biol Chem 389: 739–746 18627300 10.1515/BC.2008.085 PMC2580060

[bib38] Shaw JL, Diamandis EP (2007) Distribution of 15 human kallikreins in tissues and biological fluids. Clin Chem 53: 1423–1432 17573418 10.1373/clinchem.2007.088104

[bib39] Shaw JL, Diamandis EP (2008) A potential role for tissue kallikrein-related peptidases in human cervico-vaginal physiology. Biol Chem 389: 681–688 18627298 10.1515/BC.2008.069

[bib40] Sidiropoulos M, Pampalakis G, Sotiropoulou G, Katsaros D, Diamandis EP (2005) Downregulation of human kallikrein 10 (*KLK10/NES1*) by CpG island hypermethylation in breast, ovarian and prostate cancers. Tumor Biol 26: 324–336 10.1159/000089290 16254462

[bib41] Smith Jr RS, Gao L, Chao L, Chao J (2008) Tissue kallikrein and kinin infusion promotes neovascularizatio in limb ischemia. Biol Chem 389: 725–730 18627294 10.1515/BC.2008.084

[bib42] Sturgeon C (2002) Practice guidelines for tumor marker use in the clinic. Clin Chem 48: 1151–1159 12142367

[bib43] Talieri M, Diamandis EP, Gourgiotis D, Mathioudaki K, Scorilas A (2004) Expression analysis of the human kallikrein 7 (*KLK7*) in breast tumors: a new potential biomarker for prognosis of breast carcinoma. Thromb Haemost 91: 180–186 14691584 10.1160/TH03-05-0261

[bib44] Talieri M, Prezas P, Alexopoulou DK, Mathioudaki K, Diamandis EP, Xynopoulos D, Ardavanis A, Arnogiannaki N, Scorilas A (2009) Clinical significance of kallikrein-related peptidase 7 (*KLK7*) in colorectal cancer. Thromb Haemost (in press) 19350120

[bib45] Worsham MJ, Chen KM, Meduri V, Nygren AOH, Frrami A, Schouten JP, Benninger M (2006) Epigenetic events of disease progression in head and neck squamous cell carcinoma. Arch Otolaryngol Head Neck Surg 132: 668–677 16785414 10.1001/archotol.132.6.668

[bib46] Yousef GM, Borgono CA, Popalis C, Yacoub GM, Polymeris ME, Soosaipillai A, Diamandis EP (2004) In-silico analysis of kallikrein gene expression in pancreatic and colon cancers. Anticancer Res 24: 43–51 15015574

[bib47] Zheng Y, Cai T, Feng Z (2006) Application of the time-dependent ROC curves for prognostic accuracy with multiple biomarkers. Biometrics 62: 279–287 16542256 10.1111/j.1541-0420.2005.00441.x

[bib48] Zheng Y, Katsaros D, Shan SJ, de la Longrais IR, Porpiglia M, Scorilas A, Kim NW, Wolfert RL, Simon I, Li L, Feng Z, Diamandis EP (2007) A multiparametric panel for ovarian cancer diagnosis, prognosis, and response to chemotherapy. Clin Cancer Res 13: 6984–6992 18056174 10.1158/1078-0432.CCR-07-1409

