# Supplementary material for: The use of kallikrein-related peptidases as adjuvant prognostic markers in colorectal cancer
Source: Br J Cancer. 2009 Apr 14;100(10):1659–65. doi: 10.1038/sj.bjc.6605033 (PMC2696752; doi:10.1038/sj.bjc.6605033)
Supplement: Supplementary Figure and Table [file 6605033x1.doc]

Supplementary Data

**Table S1.** Spearman correlation coefficients between KLK markers and hierarchical clustering based on Spearman’s correlation.

|  | KLK5 | KLK6 | KLK7 | KLK8 | KLK10 | KLK11 | KLK13 | KLK14 | KLK15 |
| --- | --- | --- | --- | --- | --- | --- | --- | --- | --- |
| KLK5 | 1 | 0.203 | 0.377 | 0.363 | 0.228 | 0.195 | 0.386 | 0.411 | 0.071 |
| KLK6 |  | 1 | 0.428 | 0.616 | 0.688 | 0.291 | 0.201 | 0.275 | 0.092 |
| KLK7 |  |  | 1 | 0.511 | 0.488 | 0.201 | 0.541 | 0.348 | 0.259 |
| KLK8 |  |  |  | 1 | 0.535 | 0.437 | 0.369 | 0.359 | 0.128 |
| KLK10 |  |  |  |  | 1 | 0.386 | 0.289 | 0.338 | 0.184 |
| KLK11 |  |  |  |  |  | 1 | 0.38 | 0.355 | 0.327 |
| KLK13 |  |  |  |  |  |  | 1 | 0.526 | 0.501 |
| KLK14 |  |  |  |  |  |  |  | 1 | 0.186 |
| KLK15 |  |  |  |  |  |  |  |  | 1 |

**Figure S1.** Time-dependent ROC curve at year one for overall survival. The performance of the combination of the selected clinical factors and marker panel is corrected for overfitting.


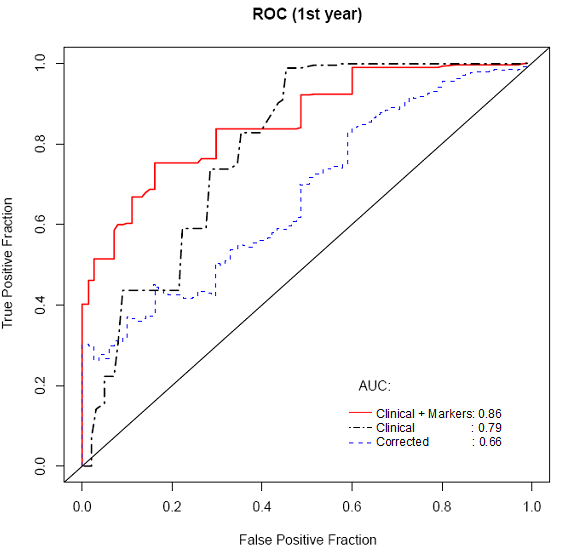


‘Clinical’: age, TNM stage and differentiation;

‘Clinical + Markers’: age, TNM stage, and KLK14;

‘Corrected’: the performance of ‘Clinical+Markers’ is corrected for over-fitting, which was estimated by cross-validation technique (2/3 of the observations as training, and remaining 1/3 as testing) randomly repeated 100 times.
